# Population Estimation and Trappability of the European Badger (*Meles meles*): Implications for Tuberculosis Management

**DOI:** 10.1371/journal.pone.0050807

**Published:** 2012-12-05

**Authors:** Andrew W. Byrne, James O’Keeffe, Stuart Green, D. Paddy Sleeman, Leigh A. L. Corner, Eamonn Gormley, Denise Murphy, S. Wayne Martin, John Davenport

**Affiliations:** 1 Teagasc, Mellows Campus, Athenry, County Galway, Ireland; 2 School of Biological, Earth and Environmental Sciences, University College Cork, North Mall Campus, Distillery Fields, Cork, Ireland; 3 School of Veterinary Medicine, University College Dublin, Dublin, Ireland; 4 Department of Agriculture, Fisheries and Food (DAFF), Ireland; 5 Teagasc Rural Economy and Development Programme (Spatial Analysis), Teagasc, Kinsealy, Dublin, Ireland; 6 Department of Population Medicine, University of Guelph, Guelph, Ontario, Canada; University of Georgia, United States of America

## Abstract

Estimates of population size and trappability inform vaccine efficacy modelling and are required for adaptive management during prolonged wildlife vaccination campaigns. We present an analysis of mark-recapture data from a badger vaccine (Bacille Calmette–Guérin) study in Ireland. This study is the largest scale (755 km^2^) mark-recapture study ever undertaken with this species. The study area was divided into three approximately equal–sized zones, each with similar survey and capture effort. A mean badger population size of 671 (SD: 76) was estimated using a closed-subpopulation model (CSpM) based on data from capturing sessions of the entire area and was consistent with a separate multiplicative model. Minimum number alive estimates calculated from the same data were on average 49–51% smaller than the CSpM estimates, but these are considered severely negatively biased when trappability is low. Population densities derived from the CSpM estimates were 0.82–1.06 badgers km^−2^, and broadly consistent with previous reports for an adjacent area. Mean trappability was estimated to be 34–35% per session across the population. By the fifth capture session, 79% of the adult badgers caught had been marked previously. Multivariable modelling suggested significant differences in badger trappability depending on zone, season and age-class. There were more putatively trap-wary badgers identified in the population than trap-happy badgers, but wariness was not related to individual’s sex, zone or season of capture. Live-trapping efficacy can vary significantly amongst sites, seasons, age, or personality, hence monitoring of trappability is recommended as part of an adaptive management regime during large–scale wildlife vaccination programs to counter biases and to improve efficiencies.

## Introduction

Infectious diseases of wild animals are rapidly becoming an emergent global issue due to their potential threats to biodiversity, agriculture and human health [1], [2], [3]. Newly emergent diseases can severely reduce wildlife populations, leading to an increased risk of species extinction (e.g. Tasmanian Devil *Sarcophilus harrisii* and facial tumour disease (FTD) [4]). Similarly, established wildlife diseases are of concern due to documented declines in threatened species (e.g. Ethiopian wolves *Canis simensis* as a result of rabies [5]). Infectious diseases in wildlife can also be problematic because of the maintenance of disease (wildlife reservoirs) within ecosystems that can affect domestic animals, humans or both [6]. In particular, bovine tuberculosis (TB), caused by the bacterium *Mycobacterium bovis*, is a globally significant disease that can affect populations of conservation concern (e.g. Lions *Panthera leo* in reserves in South Africa [7]), and maybe maintained in wild populations that are a reservoir of infection for domestic animals (badger *Meles meles* in Ireland and Britain; white-tailed deer *Odocoileus virginianus* in Michigan, USA [8]; Brushtail possum *Trichosurus vulpecula* in New Zealand [9]). The bacterium can ultimately infect humans through the consumption of animal products or direct contact with infectious hosts, and is potentially life threatening for the immunocompromised [10].

There are few effective options for managing infectious diseases in wildlife populations. Culling has been used in a number of contexts to reduce the density of diseased animals, in the anticipation that it will limit the transmission of infection within a wildlife population (intraspecific transmission) and between host species (interspecific transmission). This approach has had varying degrees of success in different animal-disease systems (see [4], [6], [11]). The effectiveness of such strategies can depend on the wildlife host’s ecology, population density, social structure, response to culling, and the reduction in population abundance achieved [12], [13]. Thus, estimates of trappability are required to assess the efficacy of culling [14]. Culling is also associated with animal welfare concerns and can be strongly opposed by public opinion, especially when the host species is of cultural significance [15], [16].

Due to these issues, vaccination has been increasingly utilised and is becoming an important tool in wildlife disease management [1]. In order for wildlife vaccination to be effective, it is essential that the target population can be reached (i.e. vaccinated). Successful vaccination programs have been implemented where the target population was reached using oral vaccine-baits (e.g. rabies in foxes *Vulpes vulpes* in Europe, reviewed in [17]). Ideally, for a vaccine strategy to be effective, the proportion of the healthy population immunized (known as vaccine coverage) should be maximised. However, if capturing the animals for vaccination is the method chosen, it may be difficult, especially if the target species is of low density, nocturnal, possibly trap-wary due to previous disturbance or exhibits variation in trappability at the individual level (bold vs. wary individuals). To conduct wildlife vaccination and management programs using capture, knowledge of the trapping biases and efficacy associated with the wildlife species of concern and trapping methodology employed are required to maximise coverage or removal efficacy [18].

Here we analyse data from a large-scale mark-recapture study for European badgers (*Meles meles*), the Kilkenny Vaccine Trial (KVT), in order to estimate population size and trappability. This vaccine trial is the first large-scale experimental BCG vaccine trial in wild badgers, and is currently the largest scale mark-recapture study ever undertaken in this species. Wildlife population sizes are difficult to estimate, especially for nocturnal species such as the badger. We employ three estimators of population size in the current study: minimum number alive (MNA), closed sub-population model (CSpM) and a simple multiplicative model (MM). All three models have been used previously to estimate badger population size (e.g. [19], [20], [21]). MNA and CSpM are mark-recapture techniques that rely on samples of the badger population prior to and after the capture session being estimated. The MM relies on the accurate identification of active setts (burrows) within the study area and estimates of social group size. We calculated the trappability estimates from each estimator as the percentage of the estimated population that was captured during a given session. The objectives of this study were to: 1. estimate the badger population size using different methods, 2. derive estimates of trappability from these estimates, 3. evaluate MNA bias compared with other estimators, 4. assess differences in capture probability amongst badger groups based on sex, age–class and trap–wariness. The implications of the findings presented in this paper will help inform the design and implementation of wildlife vaccination programs. Furthermore, the findings will be used as a baseline against which delivery systems (e.g. baits or injected vaccines) can be compared.

## Results

### Badger Captures and Recorded Fatalities

Stopped restraints were the predominant capture methodology, with 1702 captures being made by restraints, whereas 78 captures were made by cages during the study period (capture ratio: 22∶1). Cubs had significantly greater odds of being captured in a cage than other age classes (cub captures by cage = 17 vs. by restraint = 2; logistic regression p<0.001). There was no significant difference in the odds of being cage–trapped amongst the other age classes (multiple Wald tests: p>0.3). During the study period 906 unique individual badgers were captured. Of these, 2% (n = 15) were first captured as cubs and 28% (n = 258) were first captured as juveniles. Of the badgers first captured as cubs or juveniles, 27% (n = 4) and 28% (n = 72) were recaptured as adults, respectively. Overall, the recapture rate (i.e. the % of all badgers with >1 capture) was 48%, with males having higher recapture rates than females (54% and 44%, respectively; Pearson χ^2^ (DF: 1) = 9.53; P = 0.002).

Sixty-six dead badgers were recorded between the beginning of the study and April 2012; 40 of these had previously been marked. The majority of these badgers (39 badgers; 59%) were killed due to road traffic accidents (RTAs). One third (33%) of the RTA badgers had not been previously marked (13 of 39). Given the population estimates (see below), the estimated annual RTA mortality (% of total population killed) for this population is 2.0–3.3%.

### Population Size Estimates

**Figure 1:**
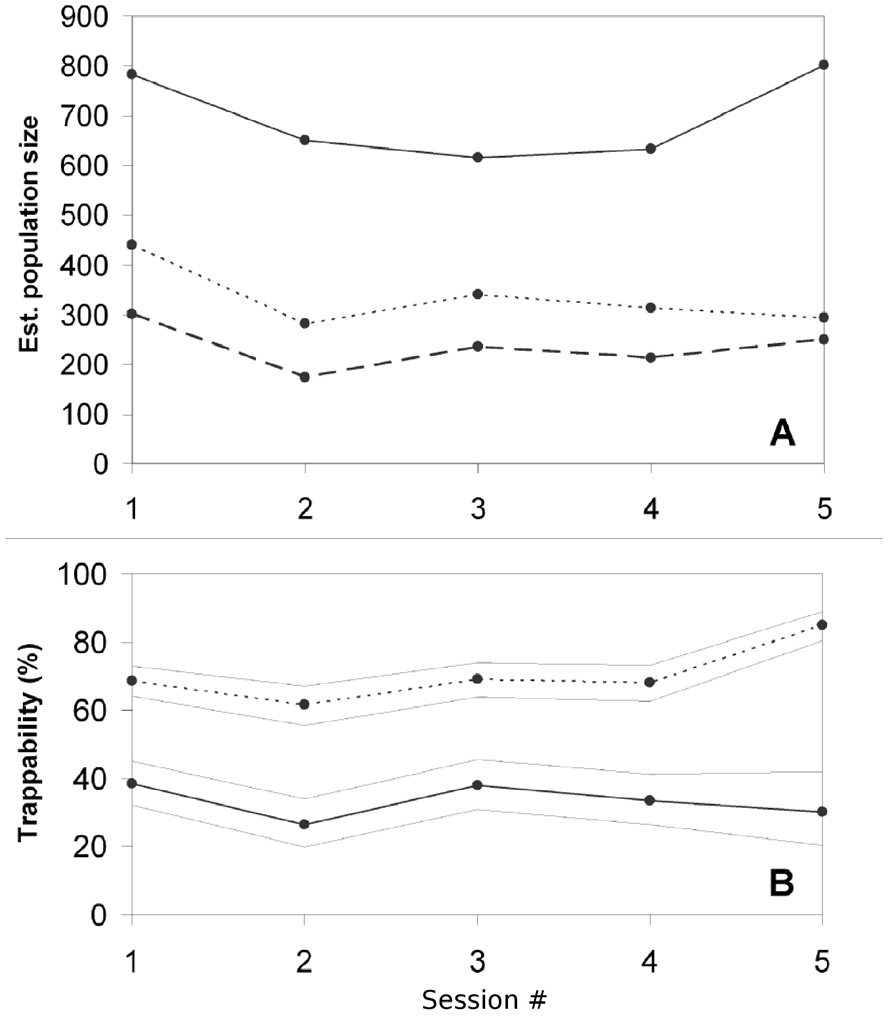
Badger population size and trappability estimates. **A.** Estimated badger population size for each full session (1–5) within the Kilkenny study area during the study period. Solid-line is the closed-subpopulation derived population estimate, the dotted line is the minimum number alive (MNA) population estimate, and the dashed line is the number of badgers trapped per session. **B.** The solid line is the estimated trappability using the closed-subpopulation model during each session with associated exact 95% confidence interval. Dotted line represents the MNA-derived trappability.

The CSpM estimates of the badger population varied from 616 badgers to 802 badgers across capture sessions, with a mean population estimate of 697 (SD 88; Figure 1A). Since the estimate of the population size during session five was potentially biased, we removed that estimate; this, reduced the CSpM mean to 671 (SD 76) badgers (Table 1). These estimates were consistent with the MM estimates of a mean population size of 676 badgers (SD 90; Table 2). CSpM estimates were always within the 95% CI of the MM (Figure S1). In comparison, the mean MNA estimate was 344 (SD 68); 49–51% smaller than the mean CSpM and multiplicative model estimates. These population estimates corresponded to densities of 0.82–1.06, 0.73–1.06 and 0.37–0.58 badgers km^−2^, for the CSpM, multiplicative and MNA models respectively.

**Table 1:** Trappability statistics and estimated population size using mark-recapture methods for each session (1–5) of the Kilkenny study area.

Session #	n	T	t	N	MNA	p_CSpM_ (95% CI)	p_MNA_ (95% CI)	MNA – N (% difference)	p_CSpM_ - p_MNA_
**0**	122								
**1**	302	224	86	783	440	38.39 (32.27–44.92)	68.63 (64.07–72.95)	−43.78	−30.24
**2**	174	148	39	651	283	26.35 (19.92–34.00)	61.48 (55.54–67.18)	−56.52	−35.13
**3**	235	169	64	616	340	37.87 (30.90–45.39)	69.12 (63.91–73.99)	−44.83	−31.25
**4**	213	150	50	633	313	33.33 (26.29–41.23)	68.05 (62.57–73.18)	−50.52	−34.72
**5**	250	63	19	802	294	30.16 (20.24–41.99)	85.03 (80.43–88.91)	−63.35	−54.87
**6**	128								
**Mean (all)**	203	151	52	697	334	33.22 (25.92–41.50)	70.46 (65.30–75.24)	−51.80	−37.24
**SD**	66			88	63	5.12	8.72	8.22	10.08
**Mean (reduced)**	235∧			671*	344*	33.99 (27.31–41.38)*	68.82 (61.52–71.83)*	−48.91*	−32.84*
**SD**	47			76	68	5.57	3.59	5.87	2.45

The captures are presented both including and excluding the potentially biased estimates of session five.

∧ excluding partial sessions 0 and 6.

* excluding potentially biased estimates from session 5.

n is the number of badgers captured; T is the closed-subpopulation; t is the number of badgers captured from T; N is the estimated population from the closed-subpopulation model (CSpM); MNA is the minimum number alive; p_CSpM_ is the trappability for each *i* ^th^ session derived from the CSpM; p_MNA_ is the trappability for each *i* ^th^ session derived from the MNA estimates; 95% CI is the exact confidence intervals for a proportion assuming no prior information.

**Table 2:** Badger numbers estimated using a multiplicative model of active main setts within the study area and estimates of badger social group sizes.

Session	Active main setts	Population size (95% CI)	Trappability (95% CI)
1	143	798 (636–971)	38% (31–47%)
2	99	553 (441–672)	31% (26–39%)
3	123	687 (547–835)	34% (23–43%)
4	114	636 (507–774)	33% (28–42%)
5	126	703 (561–856)	36% (29–45%)
Mean	121	676 (538–822)	35% (28–43%)
SD	16	90 (72–110)	2% (2–3%)

### Capture Matrix

Table 3 shows the capture matrix of badgers in the Kilkenny study area. The mean percentage of badgers captured that were marked during a previous session was 23.3% (SD 7), and the mean percentage of badgers recaptured at a subsequent session was 22.0% (SD 4). The general trend was for a smaller percentage of badgers to be shared between capture sessions the further apart these sessions were temporally. For example, sessions one and two shared 35.6% of recaptured badgers, whereas sessions one and five shared only 19.2% of recaptures.

**Table 3:** Matrix of capture percentages for sessions one to five within the Kilkenny study area.

	n	302	174	235	213	250
N	Session #	1	2	3	4	5
**302**	**1**	**100**	35.63	27.66	25.35	19.20
**174**	**2**	20.60	**100**	18.30	16.43	13.60
**235**	**3**	21.59	24.71	**100**	30.99	24.80
**213**	**4**	17.94	20.11	28.09	**100**	21.20
**250**	**5**	15.95	19.54	26.38	24.88	**100**

n is the number of badgers captured per session. Values in the upper right of the matrix represent the percentage of badgers that were recaptures from a previous session (*i*−1). The lower left of the matrix represents the percentage of badgers captured during session *i* that went on to be caught during session *i*+1.

**Figure 2:**
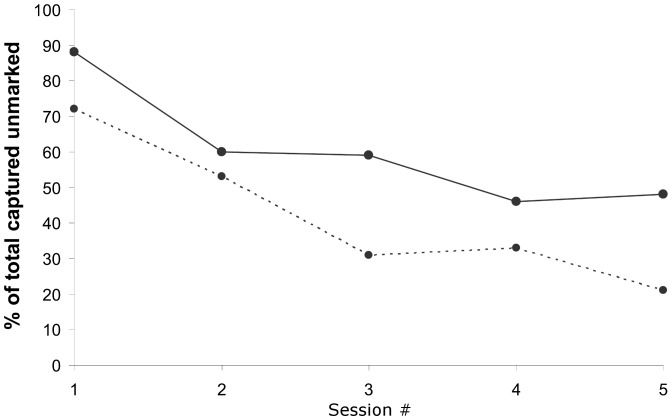
Percentage of unmarked badgers caught in a sequence of capture sessions in the Kilkenny study area. Solid line represents all badgers trapped; dashed line represents adult badgers only.

The proportion of all badgers captured that were unmarked declined from 88% to 48% between sessions one and five (Figure 2). Some of the captured badgers may have been unavailable for previous captures due to their age; hence we repeated the calculation discarding data on cub and juvenile badgers in each session. During the fifth session, 79% of the adult badgers caught had been marked previously (Figure 2).

### Trappability

The trappability estimates from the CSpM for each capture session varied between 26% and 38% (Table 1; Figure 1B) with the mean (excluding the fifth session) being 34% (SD 5). Overall, trappability using abundance estimates from MNA was significantly larger than estimates from the CSpM (p = 0.001) ranging from 61% to 85%, with a mean of 69% (SD 4; Figure 1B). Trappability was estimated for a core-only population to investigate the possible bias arising from temporary badger emigration between sessions (see Methods and Text S1). When trappability was estimated using only this core population (58% of all badgers caught), mean CSpM trappability increased marginally (by 1%) to a mean of 35% (range: 29%–41%; SD 6) for an estimate excluding the fifth session. The density estimates from this core population did not deviate significantly from that of the whole population (means: 0.91 vs. 0.92 badgers km^−2^). Trappability per session estimated from the multiplicative model was consistent with the CSpM estimate (35%; range: 31–38%; SD 2). The lower limit of population-averaged trappability (*sensu* [22]) was estimated as 30%.

A logistic mixed model suggested that capture probability was affected significantly by season and zone (p<0.05; Table 4), but not by sex or year (p>0.1). The relationship between badger age-class and trappability was dependent on the season of capture. There were higher odds of trapping a badger during autumn or winter than at other seasons, but the relative difference was significantly greater for young badgers than for adult badgers (p = 0.017; mean difference in trappability across seasons: young = 33%; adult = 6%). Also, there was a difference in trappability across zones depending on season. The significant interaction term for zone and season (p<0.01), was driven by zone C which had significantly lower trappability during the spring or summer than the other zones (mean trappability for spring/summer in zone C was 17%; mean trappability for all other zone/season combinations was 38%).

**Table 4:** Results from a logistic mixed model with random effects of the probability of a badger being trapped in the Kilkenny study area during the study period.

Model$	Odds ratio	SE	z	p
Season (autumn/winter)	54.77	62.83	3.49	<0.001
Zone A*	3.36	1.29	3.17	0.002
Zone B*	3.59	1.75	2.62	0.009
Season (autumn/winter) × ZoneA∧	0.27	0.12	−3.04	0.002
Season (autumn/winter) × ZoneB∧	0.20	0.11	−2.87	0.004
Age (adult)	2.74	1.41	1.96	0.050
Season (autumn/winter) × Age (adult)	0.25	0.14	−2.39	0.017

* Wald test of Zone A = Zone B: p = 0.96; referent Zone C.

∧ Wald test of Season (autumn/winter) x Zone A = Season (autumn/winter) x Zone B: p = 0.63.

$ Overall the model explained the variation in the dataset in comparison with a null model to a statistically significant extent (Wald χ^2^ (df: 7) = 24.3; p = 0.001), while the Hosmer-Lemeshow goodness-of-fit test indicated no statistically significant lack of fit (Pearson χ^2^ (df: 4) = 7.39; p = 0.117).

A cohort of 83 badgers was used to model the total counts of badger captures during sessions 2–4 inclusive (see materials and methods for cohort inclusion rules). In total, 49 of these badgers were caught on 90 different occasions. Individual badgers were captured 0–5 times during the period (mean: 1.08; SD 1.22). There were no significant differences in the number of captures across the sexes or age-classes. All two-way interactions offered to the model were non-significant. The final Poisson model (Table 5) indicated that there were significantly fewer captures for badgers within this cohort that was first captured in zone C than zone A (p = 0.013), but not for B (p = 0.550). Logistic models of trap wariness failed to explain the variation in the dataset in comparison with a null model (LR χ^2^ (df: 2) = 5.40; p = 0.067). Overall, there were more putatively trap-wary badgers (n = 34) than putatively trap-happy badgers (n = 13) identified in the population.

**Table 5:** Results from a Poisson model of the number of captures of a cohort of badgers known to be alive during capture sessions 2–4 inclusive in the Kilkenny study area.

Model$	Coef.	SE	Z	p
Zone A*	0.60	0.24	2.49	0.013
Zone B*	0.41	0.33	1.26	0.209
Constant	−0.26	0.19	−1.35	0.178

* Wald test of Zone A = Zone B: p = 0.55; referent Zone C.

$ Overall the model explained the variation in the dataset in comparison with a null model to a statistically significant extent (Wald χ^2^ (df: 2) = 6.52; p = 0.038).

## Discussion

### Kilkenny Badger Trappability in Context

Our study revealed a mean trappability of 34–35% per session (annual capture rate: 56–58%; calculation following [23]), as estimated from the CSpM and multiplicative models, across the entire population. A previous smaller scale study (16 km^2^) in Ireland estimated adult trappability to be 51% during the first year of trapping in a higher density (3 badgers km^−2^) badger population in east Offaly [24]. In Britain, where only cage traps were used, trappability estimates have varied across sites depending on badger density, disturbance, age–profile and seasons (Table 6; [25]). All of the study populations summarised in Table 6 had greater estimated mean trappability than our study population. However, those populations were of a much smaller size than that of our study. For example, the estimated adult population sizes was approximately 28–69 badgers in Nibley and between 180–200 for Woodchester Park and Wytham wood [19], [26], [27]. Furthermore, their study areas were smaller (6–37 km^2^) in comparison with the present study area (755 km^2^), making the recapture of a high proportion of individuals more achievable.

**Table 6:** Summary of trappability estimates from studies of the European badger from Britain gathered from published sources.

Study site	Density	Adult trappability	Cubtrappability	Average trappability	Min/max trappability	Recent disturbance	Data sources
Nibley (1995–1997)	4–8	39% (SD 21)	68% (SD 12)	46% (SD 23)	0%–89%	Yes	[25] [56]
Woodchester Park(1995–1997)	20–35	60% (SD 21)	73% (SD 13)	64% (SD 18)	23%–100%	No	[25] [56]
Woodchester Park (2008)				57% (SD 22)*	29%–100%*	No	[48]
Wytham Wood (1995–1997)	31–48	57% (SD 10)	36% (SD 16)	52% (SD 15)	13%–70%	No	[25] [56]

Density: badgers km^−2^.

* Trappability was derived from the numbers of badgers trapped as a percentage of the minimum number alive per social group.

Estimates of the population size using minimum number alive (MNA) were always significantly lower than the corresponding closed-subpopulation or multiplicative model estimates (Figures 1A, S1, Tables 1, 2). The population size underestimate (negative bias) of MNA increases with decreasing trappability [28]. Thus, in our case where trappability was medium-low, the difference was large (49–51%) between the population size estimates from the CSpM/multiplicative model and the MNA, while the difference tends to be less pronounced (∼10–20% difference) where estimated trappability was higher, such as in long-term studies in Wytham Wood, United Kingdom (UK) [20]. The technical and logistical effort required to capture large proportions of a badger population is challenging at large spatial scales, and therefore negatively biased estimates of abundance such as MNA, that may yield overly optimistic estimates of trappability, should be avoided. Indeed, some authors suggest that MNA should be employed only if a trappability of ≥70% is achieved (e.g. [28]). In the present study, mean trappability using MNA estimates were 33–37% greater than those derived from the other methods. The density estimates derived from the CSpM and multiplicative model were broadly consistent with reports from previous large-scale (252 km^2^) studies from County Kilkenny (1.08 badgers km^−2^; [29]). In contrast, the estimates from MNA were less than half the expected density for the area. However, the CSpM/multiplicative model density estimates are still low for pasture-dominated landscapes in Ireland when compared with other (albeit smaller scale) studies (1.6–6.4 badgers km^−2^; [15]) and this may reflect a reduction in abundance from past culls [30].

### Why Might Badger Trappability Vary?

Tuyttens et al [25] speculated about the possible reasons for the differing trappabilities of badgers within and across populations. They proposed that previous culling selectively removed “trap-happy” badgers, and the remaining population then being saturated with “trap-shy” badgers. They also suggested that past culling could have altered the behaviour of badgers that survived the cull. The area of Kilkenny studied was not culled for two years prior to the study start date [31]. However, it is currently unknown how long the effects of culling impacts upon badger populations after cessation in Ireland. In the present study a group of badgers was used to assess wariness and of these, there were more badgers identified as putatively “trap-shy” than “trap-happy”. This finding may give some support to Tuyttens et al. [25] hypothesis. It should be noted that individual trapping heterogeneities violate an assumption of the CSpM and MNA and this may have biased the estimates derived from these models [25], [32]. For example, there may be some badgers that are truly ‘untrappable’, and so are never recorded during a trapping study. Evidence from longitudinal trapping studies of badgers suggests that this proportion of the population may be very small [26]. In the present study, ancillary data (i.e. from RTA badgers) were used to reduce this possible bias. Individual trapping heterogeneities may have biased our mark-recapture models; however our calculations using the multiplicative model as a baseline comparison suggests that this bias was likely not to have been great.

The simplest explanation for the observed differences in trappability amongst studies, is that trappability is a function of population density (as noted in low density populations in continental Europe: [33]) and study area size. However, other factors may have affected the differing outcomes. The British study populations in Wytham and Woodchester have been trapped repeatedly (2–4 times yearly) for long periods of time (>20years), allowing badgers to become accustomed to the experience of being trapped. Capturing procedures also differed between our study and the investigations analysed by Tuyttens et al. [25]. Badgers were captured using some cage traps but principally in stopped restraints in the present study, but only cage traps (pre-baited in Woodchester; not pre-baited in Wytham) were used in the British long–term studies. Wire stopped restraints are believed to overcome some of the learned trap avoidance behaviours associated with cage traps [34]. However, wire stopped restraints are poor (by design) at capturing younger badgers, especially cubs ([29], [33], present study). Evidence from other animal systems suggests that restraints are more efficient at capturing wild animals than cages [35]. Our approach of using two capture techniques (restraints and cages) might avoid some inherent bias introduced by the trapping method employed (despite our low cub capture rate). However, if capturing cubs is desirable for vaccination, targeting suspected breeding setts with baited cage-traps would be strongly recommended.

### Implications for Vaccine Delivery

Vaccines can be delivered to wildlife either passively e.g. by baits deployed into the environment, or actively e.g. by capture and injection. Oral delivery of rabies vaccine to wild animals has been very successful [17], but currently there is no oral bait for TB vaccination of badgers and at present parenteral or intramuscular vaccines are being used which rely on captured badgers. Findings from the current study will be used as the basis for the development of vaccine strategies using either the oral or injectable vaccine.

In order for a vaccine to be effective at a population level, ‘herd immunity’ needs to be achieved. Herd immunity refers to the proportion of individuals with immunity in a given population [36], such that, once a herd immunity threshold is passed the basic reproductive number (R_0_) for the disease is reduced below one [37]. In other words, this is the fraction of a population that must be vaccinated and protected to reduce the mean number of secondary infections per infectious individual to less than one [37]. The required threshold for herd immunity within wild badger populations, in ‘real world’ situations, is unknown currently. It is however dependent on factors such as the R_0_ of the disease, the mixing within the population, the efficacy of the vaccine, and the proportion of the population already infected with *M. bovis*. Although the R_0_ of TB in badgers is low (1.2; estimate from [38]), the disease is chronic and an effective vaccination program would likely take many years before the beneficial effects would be detectable.

Low trapping success could have important implications for the efficacy of badger vaccine programs using the parenteral or intramuscular vaccine. While trappability for each session of our study was medium-low, by the final complete session 79% of adult badgers captured had been previously captured. Simulation models based on data on badgers in England suggest that a minimum of 40–50% of the healthy badger population needs to be immunized annually over long periods to eradicate TB in a badger population [39]. However, the data used for model parameterization was from high density populations so such models may not be reliable for lower density populations found in Ireland or continental Europe [40]. In terms of the vaccine study in Kilkenny, a simulation study has suggested that low recapture percentage has only a small effect on the power to detect the effect of BCG on the wild badger population [41]. In any reasonable scenario, the benefits of vaccinating badgers as a means of reducing TB in badgers and subsequently in cattle would take a long period of time before being realized [42]. If vaccine is to be delivered by injection, then monitoring trends in trappability over time will be required as part of a flexible adaptive management strategy in future long-term vaccine programs [5]. Such monitoring would permit trapping biases to be identified and counteracted. It would also help in developing strategies to maximize capture efficiencies, with benefits for both vaccination and population management strategies.

## Methodology

### Study Area

**Figure 3:**
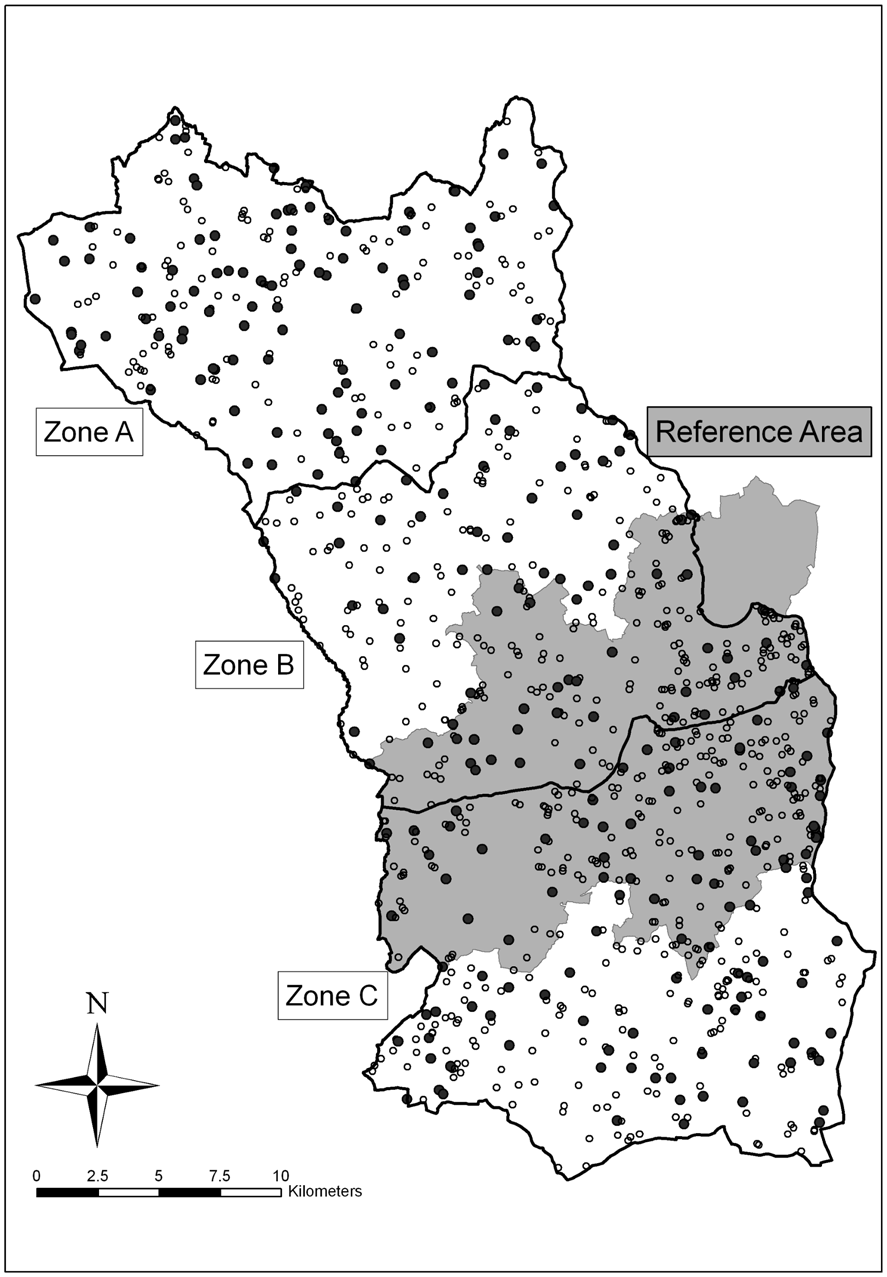
Map of the study area in Co. Kilkenny. The area is divided into three zones, A, B and C. The ‘reference area’ from the Four Area Project [12] is shaded. Dots represent all known setts (both active and inactive) within the trial area. Black dots are main setts; hollow dots are non-main setts.

The location of the study area was selected using a multi-criterion process as outlined by [31], which included previous badger-culling history, knowledge of sett locations and local technical support. The site is located in the north-west of County Kilkenny, Ireland (Figure 3) at 52.6477°N 7.2561°W. The size of the area is approximately 755 km^2^ and it is characterised by low level, rich pasture land divided by an extensive hedgerow network. Approximately one-third of this area was part of a reference area in the Four Area Project (a large scale TB-related experimental project), where culling in response to herd breakdowns was limited during the years 1997–2002 (97 badgers removed; [12]). Furthermore, the area was protected from culling for two years prior to the beginning of the vaccine trial, which began in September 2009 [31]. The site was divided into three zones (A, B and C), for the purposes of the vaccination component of the study (see [31], [43]). The three zones were matched in terms of size (228–287 km^2^), cattle densities and the number of active main setts (a type of badger burrow used most frequently within a territory, and typically the place of breeding) during initial surveys [31]. The eastern side of the study area is bounded by the River Nore which is considered to be an impediment to badger movement [29]. The remaining borders of the study areas are not considered impediments against badger movements. These borders are delineated either by roadways or small rivers, and they are more likely to define the boundary of badger territories than open country.

### Capture Protocol

The entire study area was surveyed prior to study commencement and sett locations were recorded in a geo-database. Attempts were made to capture badgers at all active setts within the trial area in a ‘session’. Typically a session lasted 20–24 weeks, depending on the length of time needed to attempt capture at all active setts. All setts were visited twice each year during an autumn/winter session (September to February) and a spring/summer session (March to July). Five complete capture sessions of the study area were conducted in total. Session one commenced in September 2009 and session five was completed in January 2012. We have also used additional smaller scale capture data collected prior to the initial full session (June 2008 – August 2009) and after the fifth session (February – April 2012); we denote these as partial sessions zero and six.

The capture of badgers was conducted under licenses (1876 Cruelty to Animals Act) issued by the Irish Department of Health & Children. Work on badgers was approved by the University College Dublin animal ethics committee. Standard badger capturing protocol was employed during this study, where traps were laid by experienced field staff in a manner which would maximise the probability of capturing a badger (for example at active burrow entrances, along badger ‘runs’, etc.). Stopped wire restraints were used to capture badgers throughout the study with cage traps used at some setts as a supplementary capture methodology. Capturing methods used conformed to national legislation for the humane trapping of wildlife (Wildlife Act, 1976, Regulations 2003 (S.l. 620 of 2003)). Cubs are more likely to be trapped in cages as their body size is too small for them to be retained in a wire restraint. Cage traps were baited daily with peanuts (but not pre-baited prior to capture attempts). During a session, each active sett was captured for an 8–night period and all traps were checked daily before 12 pm.

Captured badgers were anaesthetised with ketamine hydrochloride (0.1 ml kg^−1^) and medetomidine (Domitor®; 0.1 ml kg^−1^) administered by intramuscular injection [44]. When first captured, each badger was implanted with an identifying passive transponder and tattooed with a unique number in the inguinal region. All captured badgers were weighed and badger age was classified based on tooth wear as cub, juvenile or adult [44].

Dead badgers found at setts, on farms, or on roadsides following road traffic accidents (RTAs), were also recorded. The date, the location or nearest sett, whether it was marked (and if so, the badger’s identity) and the probable cause of death were recorded.

### Population Size

Three methods of estimating population size were employed within the study area during each capture session: a closed-subpopulation method (CSpM), minimum number alive (MNA), and a multiplicative social group estimate (MM). The CSpM is based on the Parr-Manly and Chapman methods which were developed for and applied to badgers [19], [25], [45]. This model was developed because most badger capturing strategies have a frequency of capture and capture probabilities that are lower than those required by other statistical strategies to produce reasonable population estimates (e.g. [46]). Furthermore, the experience of researchers during long-term monitoring of badger populations [20], [27], [47] indicated that other open-population statistical estimators, such as Jolly-Seber models, can overestimate badger population size. Simulation modelling suggests that CSpM is comparably accurate and precise as Jolly-Seber models, and significantly better than MNA estimates [45]. The CSpM model allows for ancillary data to be used in estimating the population size during each capture event which we denote using “*i”*. For example, in addition to the mark-recapture data, badgers that are known to be alive and within the study area (e.g. badgers marked prior to session *i* and found dead within the study area after session *i*) at session *i* can be included in the estimation. Young badgers found within one year after the *i* ^th^ trapping event were also included (following [19], [25]). We also used data on marked badgers found dead around the periphery of the study area in our calculations, under the assumption that their territories overlapped the study area. Badgers found more than 1 km beyond the study area boundary were not used.

The CSpM was derived from:
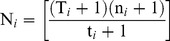

- N*_i_* is the estimated population size during the *i* ^th^ session.
- n*_i_* is the total number of badgers actually caught during session *i*.
- T*_i_* represents the (assumed) closed-subpopulation, made up of all known badgers that were alive at session *i*; badgers known to be in the area as derived from capture status (i.e. caught before and after the *i* ^th^ event), age or RTA status, and cubs that were caught later that year that were probably within the population during time *i*. To maximise the T*_i_* subpopulation, we used smaller scale badger captures (partial sessions 0 and 6) that took place within the study area prior to, and after, the five standardised sessions of the mark-recapture study.
- t*_i_* are the badgers that were caught only during this *i* ^th^ session that were part of the T*_i_* subpopulation.

All adult badgers within the T*_i_* subpopulation have at least two ‘presence’ records within the study area. Adult badgers that were captured only once were discarded from the estimates, as there was no way of ascertaining whether these badgers were residents or visitors. The CSpM methodology requires that there are sampling periods prior to and after the period that is to be estimated. Thus, an estimate of the population size for session five relied on a partial session (six), so that estimate may be biased. We present results both including and excluding estimates from session five, but mainly rely on the latter for inference. Following Tuyttens et al. [19], [25], we used the number of adult badgers captured during session two as a surrogate for badgers that were alive and available to be captured during session one. Thus, using these methods, we were able to estimate population sizes and trappability for sessions one to five. All recaptures within a session were considered a single capture, irrespective of there being multiple recaptures of individuals within each session. The average number of captures per badger within each session was 1.21 (SD 0.46); of the badgers captured per session, 80% were only captured once.

The second mark-recapture metric of population size used was Minimum Numbers Alive (MNA; [32]). While this method has been criticised for underestimating true animal population size (e.g. [28]), it has been used extensively in estimating badger populations elsewhere (e.g. [20], [26], [48], [49]). MNA was defined as:
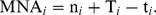



MNA*_i_* is the minimum number of badgers known to be alive at session *i*, where:

- n*_i_* is the total badgers captured within the study area during session *i*.
- T*_i_* is the total population known to be available for capture (the subpopulation) at session *i*.
- t*_i_* is the number of badgers caught from this T*_i_* subpopulation during session *i*.

The final abundance estimate was derived by multiplying a mean social group size by the number of active main setts within the study area during each session. This method has been traditionally used to estimate badger population sizes at large spatial scales (e.g. estimates for the Republic of Ireland, Northern Ireland and Britain [21], [50], [51], [52]). Mean social group size was derived from the literature and a recent review of Irish badger ecology ([15]; see supporting information Text S1). An estimate of variance (95% CI) was derived using bootstrapping with 1000 re-samples of the data (Text S1). Main sett classification was taken from the Wildlife Unit database maintained by the Department of Agriculture, Food and the Marine, Ireland. Main setts were considered active only if a badger was captured at that sett during that trapping session. This method assumes one main sett per social group territory. During all population size calculations captures from both stopped restraints and cages were pooled.

### Trappability

We used the population estimates for each session to estimate trappability (p*_i_*) for each session. Trappability estimates from the CSpM was restricted to the closed part of the population, thus for the CSpM, trappability was calculated as:
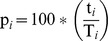



Trappability was calculated for MNA and MM estimates as the percentage of estimated total population that was captured during each session:
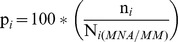



We also calculated the minimum trappability, as described by Krebs and Boonstra [22], as an estimate of the lower limit of the population-averaged trappability. The minimum trappability method ignores badgers which were captured during only one session and badgers that were captured twice during immediately successive sessions. Known-fate badgers (i.e. badgers that died during a session period) also were used in these calculations.

Badgers in rural Irish landscapes may be more mobile than higher density populations elsewhere (e.g. [53]; A. Byrne, unpublished data). Thus, there is opportunity for badgers to temporarily move outside of the study area between sessions. If this is the case, estimates of trappability and population size could be biased. To investigate this possibility, we repeated the population and trappability estimates (using CSpM) including only badgers caught initially at setts located within the study area and ≥2 km inside its boundary (a ‘core’ population; supporting information Text S1 and Figure S2). Therefore, this approach assumed that temporary movements (if made) were of distances ≤2 km, which is well supported with data from this population (A. Byrne, unpublished data). The core was comprised of approximately 60% of all known setts within the study area. We also compared the density estimates derived from this subset of data with estimates for the total area. If there was a significant difference in the density and trappability estimates between the core population and the total dataset, we would have to reject the outcomes from the models using the full dataset. Conversely, if the estimates were equivalent, we can assume that temporary emigration (as detected through our trapping records) was not a major confounder for our population estimates.

### Multivariable Models

We modelled the effects of sex, age-class (cubs and juveniles were aggregated), season (autumn/winter vs. spring/summer), year (not calendar years, but elapsed years from the beginning of the trial) and zone (zone A, B or C) on badger capture probability using logistic random effect models (*xtlogit* command in Stata®), with the badger identity as the random effect [4]. All two-way interaction terms were included in initial models and retained if they were significant predictors of trappability. To test the effect of these variables on trappability, we used only badgers that were known to be alive during the study period and assumed to be within the study area, by including only T*_i_* badgers. The fit of the logistic model was assessed using the Hosmer and Lemeshow goodness-of-fit test [54]. The ability of the model to explain variation in the dataset was assessed by comparing the final model to a null model with a likelihood ratio test.

As an alternative index of trappability, we developed a Generalised Linear Model (GLM) using the total count (including multiple captures within sessions) of captures for a group of animals that were known to be alive within the population [55]. Counts were modelled using a Poisson distribution. To maximise the badger group that was known to be alive for this analysis, and to ensure the greatest time period between the first and last captures, we retained badgers that were captured at the beginning of the study (sessions 0 and 1) and recaptured at the end of the study period (sessions 5 and 6). We assumed that these badgers were available to be trapped during the intervening trapping (2–4) sessions. Independent variables tested in the count model included sex, age-class (at first capture), zone and two-way interactions.

It is known that some badgers actively avoid capture (e.g. [34]), so we investigated trap wariness in badgers by defining a putative ‘trap-wary’ badger as one that was available to be captured during sessions 2–4 and yet was not captured. We defined a ‘trap-happy’ group, as consisting of adult badgers that were captured three times or more during session’s two to four. We used a logistic model, similar in structure to the above, to model the effects of sex, age-class, and zone and two-way interactions on the probability of an adult badger being trap-wary.

## Supporting Information

Figure S1 **Estimated population size during each capturing session of the Kilkenny vaccine trial.** The closed-subpopulation estimate (N) was always within the 95% CI of the multiplicative social group population estimate. Minimum numbers alive (MNA) were significantly lower population estimates.
(TIF)

Figure S2 **Study area in Kilkenny.** The grey area represents the areas removed from the analysis in order to estimate trappability and population density within a core area only.
(TIF)

Text S1 (DOC)
