# Supplementary figures and images for: Population Estimation and Trappability of the European Badger (Meles meles): Implications for Tuberculosis Management
Source: PLoS One. 2012 Dec 5;7(12):e50807. doi: 10.1371/journal.pone.0050807 (PMC3515448; doi:10.1371/journal.pone.0050807)

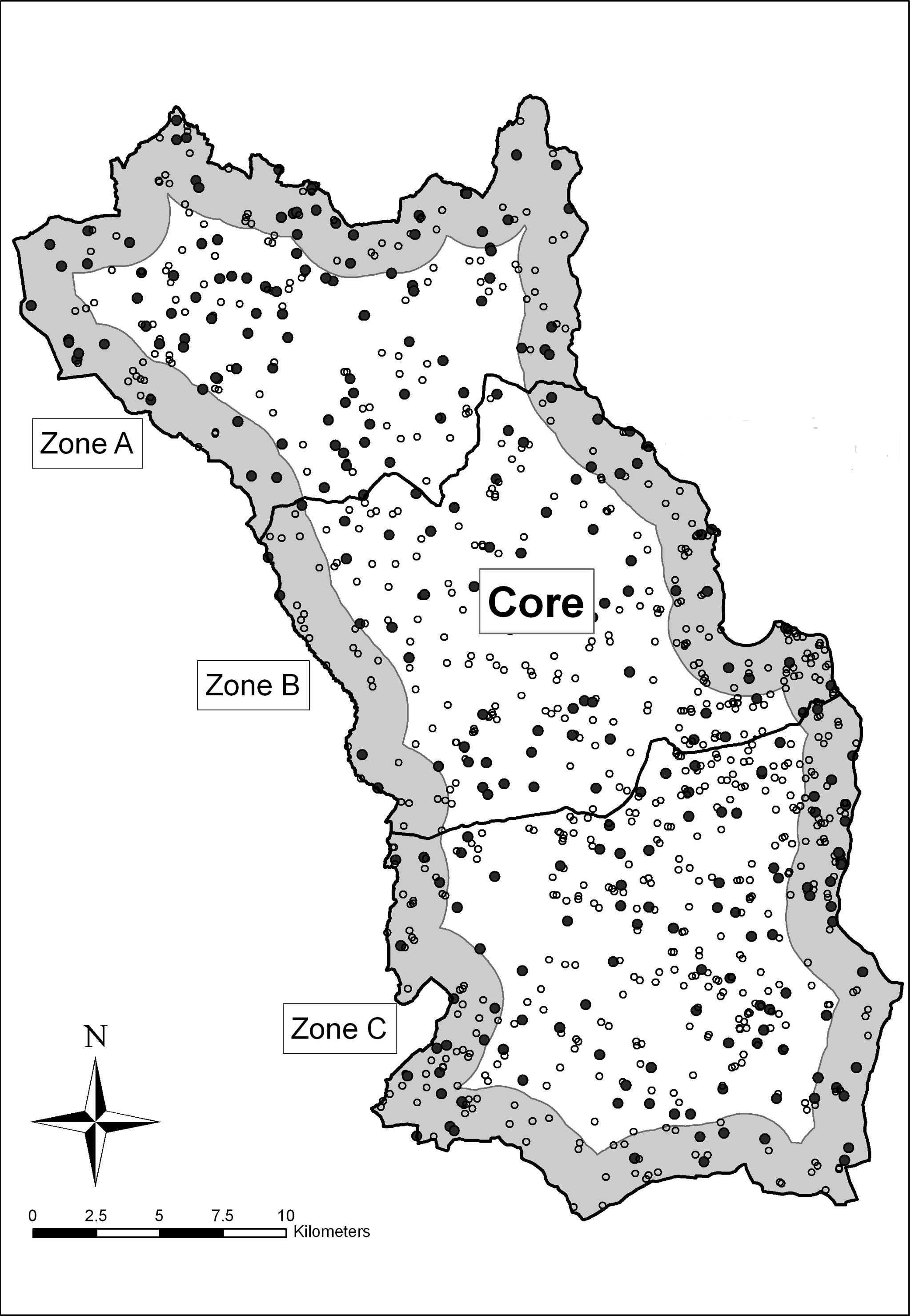

Supplement: Figure S2 — Study area in Kilkenny. The grey area represents the areas removed from the analysis in order to estimate trappability and population density within a core area only. (TIF) [file pone.0050807.s002.tif]
