# Supplementary material for: Population Estimation and Trappability of the European Badger (Meles meles): Implications for Tuberculosis Management
Source: PLoS One. 2012 Dec 5;7(12):e50807. doi: 10.1371/journal.pone.0050807 (PMC3515448; doi:10.1371/journal.pone.0050807)
Supplement: Text S1 — (DOC) [file pone.0050807.s003.doc]

**Supporting information:**

**Testing the effect of proximity to study boundary on population estimate**

To ensure that the trappability and abundance estimates were robust to the possibility of temporary emigration from the study area, an analysis of badgers only captured initially in a core area of the study site was undertaken. Only badgers first captured at setts in the core area within 2 km or more from the border of the study area were used. These badgers could be recaptured at any sett within the whole study area thereafter. The figure S2 shows the buffer used to select these setts. Four hundred and ten setts of 1009 known setts within the study area were found within this buffered area and badgers caught for the first time at any of these setts were removed from further analysis. We used the closed-subpopulation model to estimate the population size and trappability within this area. We estimated the density of badgers by dividing the estimated population size by the area of the core (454 km^2^), assuming that the core area represented the effective sampling area. These estimates are presented in Table S1.

**Mean social group size data informing the multiplicative model**

Estimates of mean badger social group size were derived from published literature on adult and social group sizes from Ireland and medium-low density populations elsewhere. We included only studies from the island of Ireland to derive adult-only group size (Table S2). Most studies from Ireland reported adult only estimates of group size. To extend our estimates to include non-adult animals, we reviewed the minimum difference in adult and social group sizes. We limited our dataset to include only social groups that reported both group sizes from Ireland and medium-low density populations in Britain and continental Europe. Therefore, we excluded high density populations where large group sizes have been reported (e.g. Woodchester Park and Wytham Wood, UK). Data were extracted from a recent review of Irish badger ecology [1], paper records or using the search term ‘badger social group’ in the online databases Google Scholar, Science Direct and ISI Web of Knowledge to identify relevant records. Group size data were subjected to 1000 bootstrap re-samples, which produced an overall mean and 95% confidence intervals. Social group size confidence intervals incorporated uncertainty around adult and social group size additively.

**Table S1:**  The numbers of badgers captured per session (n), the closed-subpopulation (T), the number of badgers caught that were part of the closed-subpopulation (t), and the estimated trappability for each session of the trial. The core area and estimated density (badgers km^-2^) of badgers present.

| **Session** | **n** | **T** | **t** | **N** | **p** | **Core area (km^2^)** | **Density** |
| --- | --- | --- | --- | --- | --- | --- | --- |
| 1 | 178 | 144 | 56 | 454 | 39 | 454 | 1.00 |
| 2 | 116 | 96 | 28 | 390 | 29 | 454 | 0.86 |
| 3 | 142 | 101 | 41 | 346 | 41 | 454 | 0.76 |
| 4 | 130 | 87 | 26 | 426 | 30 | 454 | 0.94 |
| 5 | 139 | 37 | 11 | 442 | 30 | 454 | 0.97 |
| Mean |  |  |  |  | 34% |  | 0.91 |
| SD |  |  |  |  | 6 |  | 0.10 |
| Mean (minus fifth session) | |  |  |  | 35% |  | 0.89 |
| SD |  |  |  |  | 6 |  | 0.10 |

**Table S2:** Reported mean adult badger group sizes from populations on the island of Ireland (taken from [1], with further additions). The mean adult group size across studies was used to inform a multiplicative model with active main setts. CI = confidence intervals derived from bootstrapping with 1000 re-samples.

| **Study** | **Study area** | **Adult group size** |
| --- | --- | --- |
| [2] | Sites across Northern Ireland | 6 |
| [3] | Sites across Rep. of Ireland | 5.9 |
| [4] | Offaly | 5.8 |
| [5] | Waterford | 5.5 |
| [6] | Offaly | 4.6 |
| [3] | Sites across Rep. of Ireland | 4.3 |
| [4] | Offaly | 4 |
| [7] | Cork, Kilkenny, Donegal, Monaghan | 3.9 |
| [8] | Cork | 3.8 |
| [9] | Antrim, Down | 3.8 |
| [10] | Sligo | 3.5 |
| [11] in [7] | Offaly | 3 |
| [12] | Cork, Kilkenny, Donegal, Monaghan | 2.9 |
| [9] in [7] | Antrim, Down | 2.3 |
| [13] | Down | 1.8 |
| Mean |  | 4.1 |
| Median |  | 3.9 |
| Lower 95% CI |  | 3.4 |
| Upper 95% CI |  | 4.7 |

**Table S3:** Reported minimum mean adult and social badger group sizes from Ireland and medium-low density populations in Britain and continental Europe (taken from [1], with further additions). The mean difference between adult group size and social group size across studies was used to inform a multiplicative model with active main setts. CI = confidence intervals derived from bootstrapping with 1000 re-samples.

| **Study** | **Country** | **Adults only** | **Social group** | **Difference** |
| --- | --- | --- | --- | --- |
| [9] | Ireland (Castleward) | 6.3 | 9.3 | 3 |
| [14] | Portugal | 3 | 6 | 3 |
| [15] | Britain (Avon) | 3.6 | 5.7 | 2.1 |
| [16] | Britain (Bristol) | 3 | 5 | 2 |
| [17] | Luxembourg | 2.6 | 4.6 | 2 |
| [5] | Ireland (Waterford) | 5.5 | 7.3 | 1.8 |
| [18] | Germany | 2 | 3.7 | 1.7 |
| [15] | Britain (Cornwall) | 3.3 | 4.8 | 1.5 |
| [19] | Poland (Bialowieza) | 2.4 | 3.9 | 1.5 |
| [20] | Poland (Rogów) | 2.1 | 3.5 | 1.4 |
| [21] | Spain | 3.2 | 4.6 | 1.4 |
| [22] | Belgium | 1.9 | 3 | 1.1 |
| [23] | Switzerland | 2 | 2.8 | 0.8 |
| [9] | Ireland (Glenwhirry) | 2.5 | 3 | 0.5 |
| [4] | Ireland (Offaly) | 3.5 | 3.9 | 0.4 |
| [9] | Ireland (Katesbridge) | 2 | 2 | 0 |
| Mean |  |  |  | 1.5 |
| Median |  |  |  | 1.5 |
| Lower 95% CI |  |  |  | 1.1 |
| Upper 95% CI |  |  |  | 1.9 |

**References**

1. Byrne AW, O'Keeffe J, Sleeman DP, Davenport J (2012) The ecology of the European badger (*Meles meles*) in Ireland – a review. Proc R Ir Acad B 112:105–132.

2. Kostka BI (2012) Landscape ecology, diet composition and energetics of the Eurasian badger (*Meles meles*). PhD thesis, Queens University Belfast.

3. Smal C (1995) The badger and habitat survey of Ireland. Government Stationery Office, Dublin.

4. O'Corry–Crowe G, Eves J, Hayden TJ (1993) Sett distribution, territory size and population density of badgers (*Meles meles* L.) in East Offaly. In: Hayden TJF, ed. The badger, 35–56. Royal Irish Academy, Dublin.

5. Sleeman DP, Partridge T, O’Boyle I, Gormley E, Toolan D (2010) The badgers (*Meles meles* (L.)) of Little Island, Co. Waterford. Ir Nat J 31: 94–99.

6. McGrath G (2001) The categorisation of badger habitats using Geographic Information Systems*.* Thesis, University of Dublin, Trinity College.

7. Sleeman DP, Davenport J, More SJ, Clegg TA, Collins JD et al. (2009) How many Eurasian badgers *Meles meles* L. are there in the Republic of Ireland? Eur J Wildl Res 55:333–344.

8. Sleeman DP, Mulcahy MF (2005) Loss of territoriality in a local badger *Meles meles* population at Kilmurry, Co. Cork, Ireland. Ir Nat J 28:11–19.

9. Feore S, Montgomery WI (1999) Habitat effects on the spatial ecology of the European badger (*Meles meles*). J Zool 247:537–549

10. Sleeman DP, Cussen R, Southey D, O'Leary D (2002) The Badgers *Meles meles* (L.) of Coney Island, Co Sligo. Ir Nat J 27:10–18.

11. Eves J. (1999) Impact of badger removal on bovine tuberculosis in east County Offaly. Ir Vet J 52:199–204.

12. Smal C (2002) Report to the Department of Agriculture, food and fisheries of Ireland*.* In Sleeman DP, Davenport J, More SJ, Clegg TA, Collins JD et al. (2009) How many Eurasian badgers *Meles meles* L. are there in the Republic of Ireland? Eur J Wildl Res 55:333–344.

13. Sadlier L Montgomery I (2004) The impact of sett disturbance on badger *Meles meles* numbers; when does protective legislation work? Biol Cons 119:455–462.

14. Rosalino LM, Macdonald DW, Santos–Reis M (2004) Spatial structure and land–cover use in a low–density Mediterranean population of Eurasian badgers. Can J Zool 82:1493–1502.

15. Cheeseman CL, Jones GW, Gallagher J, Mallinson PJ (1981) The population structure, density and prevalence of tuberculosis (*Mycobacterium bovis*) in badgers (*Meles meles*) from four areas in south–west England. J Appl Ecol 18:795–804.

16. Harris S, Baker PJ, Soulsbury CD (2010) Eurasian badgers (Meles meles). In Urban carnivores: 109–119. Gehrt, S.D., Riley, S.P.D. & Cypher, B.L. (Eds). Baltimore, MD: John Hopkins University Press.

17. Schley L, Schaul M, Roper TJ (2004) Distribution and population density of badgers *Meles meles* in Luxembourg. Mamm Rev 34:233–240.

18. Walliser G (2003) Auswirkungen anthropogener landnutzung auf die siedlungsstruktur, raum– und habitatnutzung des europäischen dachses (*Meles meles* L., 1758) auf der Insel Rügen*.* Dissertation (Doctor rerum naturalium). Technischen Universität Dresden, Germany.

19. Kowalczyk R, Zalewski A, Jedrzejewska B, Jedrzejewski W (2003) Spatial organization and demography of badgers (*Meles meles*) in Bialowieza Primeval Forest, Poland, and the influence of earthworms on badger densities in Europe. Can J Zool 81:74–87

20. Goszczynski J, Skoczynska J (1996) Density estimation, family group size and recruitment in a badger population near Rogów (Central Poland). Miscell Zool 19:27–33

21. Revilla E, Palomares F (2002) Spatial organization, group living and ecological correlates in low–density populations of Eurasian badgers, *Meles meles*. J Anim Ecol 71:497–512.

22. Venderick A (2007) Le Blaireau Europeen (Meles Meles, L 1758) En Region Wallonne: regards sur son éco–éthologie et estimation de sa population via la technique de l'affût*.* Licenciée en Sciences Biologiques. Université De Liège, Belgium.

23. Do Linh San E, Ferrari N, Weber JM (2007) Socio–spatial organization of Eurasian badgers (*Meles meles*) in a low–density population of central Europe. *Canadian Journal of Zoology* **85**, 973–984.
